# The Provision of Pure Drinking Water for Troops on the March

**Published:** 1900-08-25

**Authors:** 


### THE PROVISION OF PURE DRINKING WATER FOR TROOPS ON THE MARCH.

A good deal of attention was given by the section dealing with military medicine at the Paris Congress to questions relating to the prevention of typhoid fever and dysentery among troops on active service. The maintenance of cleanliness as to food, drink, air, and soil, with the immediate cremation or disinfection and burial of all dejecta were the main points insisted on. It was admitted, however, that dominating the whole problem was that of providing a pure drinking water, and it is to be confessed that, although a certain amount of hopefulness was expressed in regard to the future of chemical disinfection and purification, the drift of the discussion was to emphasise the very great difficulties which stand in the way of the extemporaneous purification of any water supply that has once been exposed to risks of contamination. The greatest stress was laid upon the advantages of boiling, but it was pointed out that boiled water is not readily taken except in the form of tea or coffee; and it was held that, although of all methods of purification it was the best for individuals or for small parties, it became almost impracticable in the field and for large armies. Filtration by the *bougie Chamberland* was stated to give good results in most of the French barracks, but even there only by the exercise of constant supervision and minute care, and the same with the Berkefeld and Maillé filters; they were equally recommendable, but their size, their fragility, and their small yield were, it was stated, opposed to their use in the field, while charcoal filters were distinctly dangerous in consequence of their liability to become infected. The chemical methods of purifying water seemed the most hopeful, and Surgeon-Major Lapasset said that the possibility of thereby obtaining extemporaneously large quantities of potable water rendered their study particularly interesting. He mentioned, in this regard, certain processes based on the employment of alum, of lime, and of perchloride of iron, all of which, however, involved a slow precipitation and then a decantation or infiltration of the liquid, which rendered them of only occasional utility for the purpose they were considering. More rapid and, therefore, more efficacious were the processes which depended upon the employment of permanganate of potassium, any excess of which could be removed by various substances, such as coffee, sugar, &c. Peroxide of chlorine also seemed to have a future, the gas being easy to prepare, soluble in water in all proportions, non-toxic, and apparently possessed of considerable sterilising energy. Surgeon-major Schucking advised for troops in the field the use of hypochlorite of calcium preceded by a simple clarifying filtration, and for establishments the introduction of the Berkefeld-Nordtmeyer filters. Dr. Vincent insisted on the necessity of sterilising the tubes of the Berkefeld or the Pasteur filters every few days, which was not easy to do in the field, and held that if these filters were to give good results constant supervision was required. Among the chemical processes which he mentioned with special approval were those based on the use of permanganates, and one founded on the employment of bromine. On the whole there seemed a general consensus to the effect that chemical purification was the coming process for use in the field, but, as was pointed out by Dr. Lapasset, much of the efficiency of all such methods of purification depended upon the water being originally in a state of comparative purity, for, as he said, it must always be very difficult to purify and deliver fit for immediate use a water which, to begin with, is muddy or very rich in organic matters. In any case he insisted that the purification of the water for use by the troops should be in the hands of a responsible officer, or, better still, of a medical man.

